# Loss of quality of corneas stored in hypothermic preservation media during processing and evaluation in an eye bank: a cohort study

**DOI:** 10.1007/s10561-026-10240-4

**Published:** 2026-07-24

**Authors:** Felipe Machado Mota, Marcos Antonio Ferreira Júnior, Christiana Velloso Rebello Hilgert, Oleci Pereira Frota, Isabelle Campos de Azevedo, Viviane Euzébia Pereira Santos, Matilde Delmina da Silva Martins, Gustavo Moura Maidana, Bethânia Karoline Alvaro Menezes, Bruna Dias Abes, Sara Raquel Pinto Borges, Guilherme Parreira Neves

**Affiliations:** 1 Federal University of Mato Grosso do Sul, UFMS, Mato Grosso Do Sul, Campo Grande, Brazil; 2 Anjos da Visão” Eye Bank of the Beneficent Association of Campo Grande - Santa Casa, Institute of Vision of Mato Grosso Do Sul, Campo Grande, Brazil; 3 Federal University of Rio Grande Do Norte, UFRN, Natal, Rio Grande Do Norte Brazil; 4 Bragança Polytechnic University, Live Well/IPB, Bragança, Portugal

**Keywords:** Eye Banks, Corneal transplantation, Epidemiology, Quality control

## Abstract

This study aimed to analyze factors associated with the loss of quality of corneas stored in hypothermic preservation media during processing and evaluation in an eye bank. A cohort study was conducted using records from 422 donors (844 corneas) between January 1, 2022, and March 31, 2024. Corneal quality was determined through slit-lamp biomicroscopy based on 13 morphological criteria, with tissues classified on a Likert-type scale from 0 (excellent) to 4 (unacceptable). For analysis, corneas were grouped as higher quality (scores 0–2: excellent, good, and fair) and lower quality (scores 3–4: poor and unacceptable). The outcome was defined as progression from higher to lower quality between the first and second evaluations. No statistically significant associations were found between donor clinical or sociodemographic variables and progression to lower quality. The relative risk (RR) of progression to the lower-quality group according to the storage medium (Optisol-GS vs Eusol-C) was low, RR 1.01 (95% Confidence Interval: 0.94 – 1.07), possibly due to the short interval between evaluations (≤ 4 days). However, a significant trend toward higher scores was observed during the second evaluation. These findings suggest that donor characteristics, processing factors, and the preservation medium did not significantly influence the deterioration of corneal quality within the analyzed period. The differences observed between the evaluations are possibly related to the subjective nature of slit-lamp assessment rather than true tissue degradation.

## Introduction

The cornea is one of the main components of the optical system and represents the most transplanted human tissue worldwide (Gain et al. 2016). High success rates in corneal transplants are largely attributed to their immunological privilege (Clahsen et al. 2023). However, to achieve these results, it is essential to consider a range of factors, from tissue procurement methods to the techniques used in surgical procedures (Cruz et al. 2021).

With the evolution of keratoplasties, this procedure has become the treatment of choice for conditions where corneal transparency is severely compromised, with no possibility of reversal through less invasive means (Foster et al. 2008). Recent estimates indicate that blindness affects at least 36 million people worldwide (Chen et al. 2024), while corneal opacity is the fifth most common cause of blindness (World Health Organization 2019).

The consolidation of keratoplasties and their high success rates have driven the demand for this type of procedure. However, cultural and religious factors (Pshea-Smith et al. 2024), coupled with logistical, structural, and screening barriers for tissue qualification, make the number of donations insufficient to meet population needs (Deshmukh et al. 2025). These limitations contribute to a global shortage of corneas, with roughly one available cornea for every 70 required (Gain et al. 2016).

Since the first successful keratoplasty was performed by Eduardo Zirm in 1905 (Von Hippel 1888), numerous advancements have transformed this procedure. One of the most significant was the discovery that corneoscleral rims stored in sealed containers at low temperatures (4 ºC) could preserve the viability of corneas (Filatov and Sitchevska 1935). This advancement was made possible by the effect of hypothermia, which prolongs the lifespan of endothelial cells and ensures better conditions for tissue use (Souza and Barretto 2017).

This discovery revolutionized the corneal donation and transplantation process by demonstrating that tissues could be procured and stored after the donor’s death. This advancement paved the way for the establishment of the first Eye Banks (EBs), institutions responsible for the procurement, management, and quality control of corneal tissues to ensure their preservation and availability for transplantation procedures (Lysakowski et al. 2022).

EBs play a fundamental role in the corneal transplant chain, and it is estimated that approximately 30% of the tissues retrieved are disqualified for transplantation after the screening performed by these institutions (Gain et al. 2016). In addition to ensuring the microbiological safety of the tissue through donor serological testing, EBs are essential for assessing tissue quality, determining which corneas are suitable for use in keratoplasties (Acharya et al. 2019).

Within this context, one of the most commonly used techniques in this process is slit-lamp biomicroscopy, which consists of a qualitative evaluation of the morphological aspects of the tissue. However, it is permeated by evaluator subjectivity, which may lead to biases and result in different assessments of the same tissue by different professionals (Cruz et al. 2022).

As this is a complex process, several factors may be associated with the loss of quality of corneas retrieved and stored in hypothermic preservation media used by EBs. These factors may be inherent to the donor, arise from corneal processing, or be related to the subjective aspects of slit-lamp evaluation performed in these institutions. Nevertheless, the actual impact of these variables on corneal quality loss is still not fully understood.

Based on these considerations, this study aims to analyze the factors associated with the loss of quality of corneas stored in hypothermic preservation media during the processing and evaluation stages in an EB.

## Methods

This is epidemiological research conducted through a cohort study using secondary data obtained from the corneal tissue procurement process records of the EB in the state of Mato Grosso do Sul, Brazil. To support the structuring of this scientific report, the checklist from the Strengthening the Reporting of Observational Studies in Epidemiology (STROBE) initiative was adopted (Von Elm et al. 2007).

The study was conducted in collaboration with the State Health Department, specifically at the State Transplant Center of Mato Grosso do Sul, as this entity holds the legal responsibility for donor records following the procurement and processing of organs and tissues for transplantation. Data were accessed for research purposes between 29/07/2024 and 13/09/2024. The records analyzed corresponded to the period from 01/01/2022 to 31/03/2024.

The chosen time frame was justified to ensure an adequate sample size, thereby guaranteeing the precision of statistical inferences and minimizing the risk of a Type II error (false negative), which occurs when an effect is falsely deemed nonexistent. Additionally, by collecting data over more than two years, potential seasonal biases related to the donation and procurement process, as well as variations in corneal classification and stratification by different evaluators, could be controlled.

The cohort population corresponded to the census of eligible cases, encompassing all records of cornea donors who met the inclusion criteria during the study period. Sample size calculation was performed using the G*Power software (Heinrich Heine University Düsseldorf, Düsseldorf, Germany).

After defining corneas from donors as the unit of analysis, the calculation was carried out considering the following parameters: a two-tailed test, an estimated effect size (odds ratio) of 1.4, Pr(Y = 1|X = 1) under H0 of 0.20, an alpha error of 0.05, a power of 0.80, and an R^2^ of other predictors ranging from 0 to 0.99. Based on these assumptions, the required sample size was calculated to be 442. Accounting for a potential 20% attrition, the final estimated sample size for the study was 531 corneas.

Donor records were included in the sample if they had been registered by the EB, had the physiological mechanism of death confirmed as either cardiopulmonary arrest (CPA) or brain death (BD), regardless of sex or age, and had undergone corneal tissue evaluation at two distinct time points.

As this study used retrospective data collected over a period of just over two years, a temporal matching criterion was adopted to ensure comparability between cases. Considering the maximum cold storage time recommended by the manufacturers of the preservation solutions used in the service (Optisol-GS, Bausch & Lomb Surgical, United States; and Eusol-C, Alchimia, Padua, Italy), which is up to 14 days, corneal follow-up was limited to this period, restricting the analysis to the 0–14-day timeframe.

The cohort baseline (T0) was defined based on the preliminary assessment of the ocular globe, performed by the EB staff immediately after tissue arrival, while still maintained in a moist chamber (gauze soaked in 0.9% saline solution) and before complete processing. At this stage, the same morphological aspects considered after excision are evaluated: epithelium exposure, epithelial defect, subepithelial opacity, stromal edema, stromal striae, stromal infiltrate, Descemet’s membrane folds, endothelial cell loss, endothelial guttata, senile arc, pterygium, scars, and specular reflection. It is at this stage that EB professionals perform an initial screening to determine the potential viability of the tissue for subsequent evaluation steps. In cases of extensive degradation, this is promptly discarded and therefore not used for transplantation.

At this initial stage, all corneas disqualified during the pre-assessment were excluded from the study to eliminate prevalent cases of ineligible tissues from the outset and thereby avoiding potential bias, as these tissues do not undergo the processing stage. The median time from death to the evaluation of the globe was 6 h 46 min (interquartile range [IQR]: 4 h 45 min–9 h 29 min). Following ocular globe processing, the corneas were placed in a hypothermic preservation solution and stored under refrigeration until the first tissue quality evaluation. When feasible, the first evaluation was performed immediately after processing; otherwise, the preserved corneas remained refrigerated until evaluation. The median time between processing and the first corneal evaluation was 1 h 0 min (IQR: 1 h 0 min–1 h 0 min). This apparent lack of dispersion is explained by the fact that most records were concentrated around the 1-h mark due to the routine recording practices of the EB.

The second evaluation (T2) was conducted later to reassess tissue quality before decision-making regarding its destination (therapeutic use or disqualification). The median time between T1 and T2 was 44 h 48 min (IQR: 33 h 0 min–63 h 30 min). Due to the corneal storage protocol under refrigeration, before each evaluation (first or second), EB professionals removed the glass vial containing the cornea immersed in the preservation solution from refrigerated storage and allowed it to equilibrate at room temperature for at least 10 min. This step was performed to minimize glass condensation and enhance visualization of the corneal tissue during slit-lamp biomicroscopic assessment.

The classification system used by the service professionals (technicians and ophthalmologists) is based on the assessment of 13 morphological criteria using slit-lamp examination. Box 1 describes the parameters evaluated for each of these criteria, which are used by the EB to determine corneal quality classification.

**Box 1:** Morphological parameters assessed using slit-lamp examination for corneal quality classification

Epithelium exposure	The loss of surface brightness, opacity, and rough appearance of the tissue is assessed. In addition, areas of drying or irregularity on the corneal surface are also evaluated
Epithelial defect	Areas that do not reflect light uniformly, appearing as depressions or irregularities on the surface, are assessed
Subepithelial opacity	Localised areas of cloudiness or haze in the anterior portion of the corneal stroma, just beneath the epithelium, are assessed
Stromal edema	Corneal thickening is assessed, along with the presence of diffuse or localised cloudiness. In more severe cases, the presence of microcysts or epithelial bullae is also evaluated
Stromal striae	The presence of fine lines, typically white or grey, extending vertically through the stroma is assessed
Stromal infiltrate	A focal area of whitish or greyish opacity within the stroma is assessed. The presence of an oedematous halo around the infiltrate is also evaluated
Descemet’s membrane folds	The presence of fine, parallel, or interlaced lines extending across the cornea is assessed
Endothelial cell loss	An indirect assessment is made through the absence or irregularity of the specular reflex on the endothelial surface, as well as the size and morphology of endothelial cells, indicating areas of probable damage or cell loss
Endothelial guttata	The presence of small protrusions or dark spots on the posterior corneal surface, visible using the specular reflection technique, is assessed. Additionally, an "orange peel" or "hammered" pattern on the endothelial surface is observed
Senile arc	The presence of an opaque ring, concentric to the iris and separated from the limbus (sclero-corneal junction) by a clear band of cornea, is assessed
Pterygium	The presence of fibrovascular growth of the conjunctiva over the cornea is checked, appearing as a triangular lesion invading the corneal limbus, most commonly in the nasal or temporal area
Scars	Areas of opacity of varying sizes, shapes, and densities are assessed. In addition, the depth of the scar (epithelial, subepithelial, stromal, or involving all layers) and its density are evaluated
Specular reflection	The sharpness and uniformity of the specular reflex are assessed

To determine the results for each evaluated criterion, EB professionals use a Likert-type scale to report ordered categorical responses based on qualitative judgment, allowing subjective assessments to be translated into structured numerical categories (Likert 1932). In the context of slit-lamp biomicroscopic evaluation, EBs in Brazil commonly adopt this approach to support corneal quality assessment and guide decision-making regarding tissue utilization, although a certain degree of subjectivity is inherently expected.

In this analysis, each observed morphological aspect is graded according to the severity of the alterations identified by the EB professionals: a score of 0 indicates the absence of alteration (criterion observed under ideal conditions); a score of 1 corresponds to mild alterations; a score of 2 to moderate (fair) alterations; a score of 3 to marked alterations; and a score of 4 to severe alterations, reflecting greater deterioration in tissue quality.

Following this evaluation, the professionals assign an overall classification of tissue quality ranging from 0 to 4, which indicates the corneal suitability for transplantation. Grades 0 and 1 are considered tissues of "excellent" and "good" quality, respectively. In contrast, corneas classified as 2 are considered "fair," those classified as 3 are regarded as "poor," and finally, tissues classified as 4 are deemed unacceptable for keratoplasty. This classification corresponds to a general assessment of corneal quality intended for transplant eligibility in a broad sense, without distinction between specific types of keratoplasty.

It is important to note that, over the study period of just over two years, seven evaluators (one EB technician and six ophthalmologists) participated in the corneal assessments. As an inherently qualitative method, slit-lamp biomicroscopy depends on the examiner’s visual interpretation of morphological criteria and their severity. Although all evaluators followed the same standardized assessment protocol, the classification of each parameter depends on individual judgment, clinical experience, and perception of tissue characteristics. Therefore, there may be a degree of variability between the first and second evaluations that reflects interobserver differences rather than true changes in tissue quality**.**

The criterion used to define the study groups was the hypothermic storage medium in which the tissues were preserved after processing. Corneas stored in Optisol-GS (Bausch & Lomb Surgical, Inc.) were classified as the reference group, as this solution is widely considered the standard hypothermic storage medium according to the scientific literature (Gimenes et al. 2022). Conversely, tissues preserved in Eusol-C (Alchimia, Padua, Italy), used as an alternative in the service, were classified as the comparison group in the cohort. It is important to highlight that, for each donor, both corneas (right and left) were stored in the same preservation medium, in accordance with the standard procedures of the EB.

After defining the reference group and the comparison group, the overall scores assigned to the tissues at the EB at first evaluation (T1) and second evaluation (T2) were grouped. Two categories were created: those classified as 0, 1, and 2 (corneas evaluated as excellent, good, and fair) and those classified as 3 and 4 (corneas evaluated as poor and unacceptable), thus dichotomising the ordinal variable.

Corneas classified as poor/unacceptable were grouped and considered as those of lower quality, while those classified as excellent, good, or fair were included in the higher-quality group.

The outcome group was defined as those corneas initially classified as excellent, good, or fair during the T1 that were reclassified as poor/unacceptable in the T2. Thus, corneas that showed progression to lower quality classifications comprised the outcome group, while those that maintained their initial classification were considered as having no outcome.

The original Likert scale has five points, with the "fair" classification occupying an intermediate position between tissue approval and rejection for therapeutic use. To determine the group in which these tissues should be included, an analysis of the proportion of utilization of these tissues was conducted. It was found that approximately two-thirds of the corneas classified as fair, both right and left, were used in optical or tectonic keratoplasties. Based on this criterion, fair corneas were grouped with those classified as excellent and good, as they are predominantly intended for transplantation.

Corneas initially classified as fair that were ultimately not used for transplantation were mainly excluded due to factors related to tissue quality or to circumstances such as cancellation or impossibility of performing the scheduled keratoplasty. In these situations, when the tissue was returned to the EB, reallocation to another recipient often became unfeasible due to the limited remaining preservation time available for storage.

**Fig. 1:**
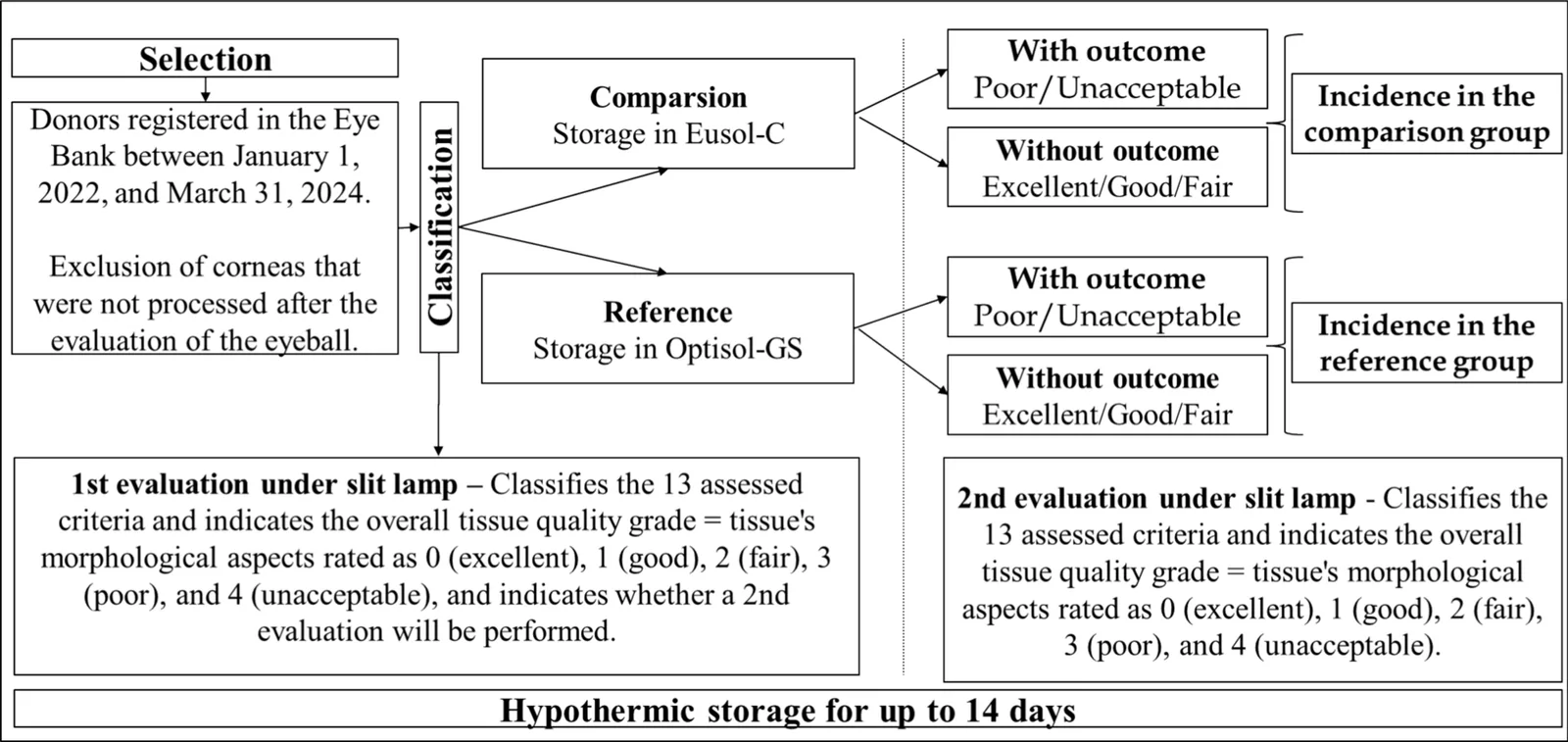
Architecture of the cohorts. Campo Grande, MS – Brazil, 2025

This categorisation is fundamental for calculating the relative risk, as it allows the determination of the incidence of corneas that progressed to the lower quality group (outcome) among those stored in Eusol-C (comparison group) and those stored in Optisol-GS (reference group). Figure 1 illustrates the structure used to compose the cohort study.

The variables collected in this study were: 1) Donor sociodemographic data: age, location of corneal tissue enucleation, sex, race/skin color, and cause of death. 2) Enucleation data: interviewer, enucleation professional, removed globe, thermal box temperature upon departure from the EB, temperature at the enucleation site, and temperature upon return to the EB. 3) Processing data: professional responsible for processing (removal of the cornea and sclera from the ocular globe), preservation medium, and body refrigeration. 4) Corneal morphological conditions: the presence of epithelial exposure, epithelial defect, subepithelial opacity, stromal edema, stromal striae, stromal infiltrate, Descemet folds, endothelial cell loss, Guttata, senile arc, pterygium, scars, and specular reflex.

The data collection form was adapted from the instrument by Cruz (2020). To ensure uniformity and the ability to encompass the desired variables, a pilot form was applied to 10% of the sample by two research team members. This preliminary assessment was conducted to pre-test and adjust the instrument. Once finalized, the material was digitized into an electronic form using Microsoft 365® and made available in the cloud, with restricted access to team members responsible for data handling and statistical analysis.

Data tabulated in electronic spreadsheets using Microsoft Excel® were analyzed with the statistical software JAMOVI® (version 2.6.13–0; The jamovi project, Sydney, Australia). Descriptive analyses (absolute and relative frequencies, measures of central tendency and dispersion) and inferential analyses (bivariate statistical tests) were performed to evaluate how the independent variables, including sociodemographic factors, donor clinical conditions, enucleation data, processing data, and corneal evaluation data, are related to the outcome variable, tissue quality classification.

The Shapiro–Wilk test and Q-Q plots were used to verify the normality distribution of the quantitative variables. Regarding the bivariate analysis to verify the association between independent variables and the dependent variable, Pearson’s Chi-Square test and Fisher’s exact test were used.

As a measure of association strength, the relative risk calculation was applied to determine to what extent the classification of corneal tissue, based on the scores assigned by professionals in both evaluations, could be influenced by the storage medium in which the tissue was preserved at the EB. Additionally, the serial binomial correlation was used as an effect size measure for the Wilcoxon test for paired samples.

As each donor may provide two corneas with distinct characteristics, inferential analyses of clinical and sociodemographic variables were performed separately for each eye (right and left). Corneas were not grouped for these specific analyses to avoid artificially inflating the sample size, since two corneas from the same donor do not correspond to two independent individuals. As the results were similar between both sides, only the p-values referring to the left corneas were presented.

For the remaining variables related to tissue processing and corneal morphological aspects, each cornea was considered an independent analytical unit. Therefore, the p-values presented refer to the total set of 844 tissues analyzed in the study.

Finally, to assess whether the tendency toward higher scores between T1 and T2 persisted regardless of the storage medium used, a stratified analysis was performed using the Wilcoxon signed-rank test for paired samples, considering the reference group (Optisol-GS; n = 568) and the comparison group (Eusol-C; n = 276).

This study received prior approval from the Research Ethics Committee for Human Subjects at the Federal University of Mato Grosso do Sul, via the Plataforma Brasil system, under Certificate of Ethical Appreciation number 78098324.3.3002.0134 and approval opinion number 7.180.512. Moreover, the Ethics Committee formally waived the requirement for obtaining informed consent.

It is important to emphasize that the study was conducted in accordance with Brazilian regulations governing research involving deceased individuals and that the data used did not originate from individuals deprived of their liberty.

## Results

During the study period, the medical records of 509 individuals were analyzed. After applying the inclusion and exclusion criteria, a total of 422 donor records were collected. From these donors, 844 corneas were processed, stored in Eusol-C or Optisol-GS, and classified regarding tissue quality at both evaluation points (T1 and T2).

When analyzing the sociodemographic characteristics of these individuals, a higher frequency of middle-aged individuals was observed, with an IQR of 59 years (43–68.8), with a minimum age of 2 and a maximum of 80 years. Regarding sex, it was found that 64.7% of the sample consisted of men. As for race/color, white and brown individuals predominated, representing 47.6% and 45.3% of all donors included in the study, respectively.

Regarding the clinical aspects related to the physiology of death, it was observed that deaths due to cardiopulmonary arrest (81.5%) were predominant. Concerning the cause of death, clinical causes (72.1%) were the most frequent.

In the bivariate analysis conducted to identify possible associations between the independent variables and the differences in assessment between the first and second evaluations performed by EB professionals, it was found that, regarding clinical and sociodemographic variables, there was no statistically significant association with the progression of tissues to a lower classification (from excellent/good/regular to poor/unacceptable) for either the right or left eyes (p > 0.05) in most variables.

The only exception was the variable "age" for the left eye (p = 0.035); however, the biserial correlation indicated a low effect size (-0.17), suggesting that the influence of this variable in the analyzed cohort is limited (Table 1).

**Table 1:** Clinical and sociodemographic characteristics of cornea donors processed by the Eye Bank. Campo Grande, MS—Brazil, 2025 (n = 422)

Variables	Donors	Corneal classification		*P*
		Progression to a lower classification	No progression to a lower classification	
Age (median, IQR)	59 (43–68.8)	–	–	**0.035**^¶^
	n* (%)			
*Sex*				0.812^*^
Female	149 (35.3%)	55	243	
Male	273 (64.7%)	82	464	
*Color/Race*				0.923^**^
Asian	03 (0.7%)	00	06	
White	201 (47.6%)	59	343	
Indigenous	01 (0.2%)	00	02	
Brown	191 (45.3%)	69	313	
Black	24 (5.7%)	09	39	
Not informed	02 (0.5%)	00	04	
*Physiology of death*				0.453^*^
Cardiac arrest	344 (81.5%)	118	570	
Brain death	78 (18.5%)	19	137	
*Cause of death*				0.933^**^
Clinical	304 (72.1%)	99	509	
Traumatic	114 (27.0%)	38	190	
Not informed	04 (0.9%)	00	08	

n*, Number of donors by classification; IQR, Interquartile Range; *, Chi-square test; **, Fisher’s exact test; ^¶^, Mann–Whitney U test

Bold values indicate statistical significance

The time between death and enucleation showed a median of 3 h and 40 min (IQR: 2 h and 24 min to 5 h and 40 min) for the Optisol-GS group and 4 h (IQR: 2 h and 35 min to 5 h and 45 min) for the Eusol-C group. The time between death and preservation showed a median of 6 h and 40 min (IQR: 4 h and 40 min to 9 h and 15 min) in the Optisol-GS group and 6 h and 50 min (IQR: 4 h and 42 min to 9 h and 21 min) in the Eusol-C group. The time between enucleation and preservation showed a median of 2 h and 30 min (IQR: 1 h and 40 min to 3 h and 51 min) for the Optisol-GS group and 2 h (IQR: 1 h and 30 min to 3 h and 30 min) for the Eusol-C group.

The median time between the first and second evaluation was 43 h and 18 min (IQR: 31 h and 30 min to 56 h and 18 min) for corneas stored in Optisol-GS, and 48 h and 36 min (IQR: 38 h and 6 min to 72 h and 42 min) for those stored in Eusol-C. Therefore, for tissues stored in Optisol-GS, the second evaluation took place within 0 to 3 days after processing, whereas for tissues stored in Eusol-C, this interval ranged from 0 to 4 days.

Due to operational aspects of the service, there was a difference in the distribution of corneas stored in Eusol-C and Optisol-GS. Of the 844 corneas processed by the EB, 276 were stored in Eusol-C, while the remaining 568 were preserved in Optisol-GS. This distribution resulted in an approximate ratio of 1:2.1 between Eusol-C and Optisol-GS.

The analysis of the relative risk of progression of corneal tissues to the lower quality group between the first and second evaluations, according to the storage medium, revealed an extremely low probability of change up to the fourth day. The relative risk was 1.01 (95% CI: 0.94–1.07).

Considering both right and left corneas together, 132 of 844 tissues (15.64%) initially classified as high quality (excellent, good, or fair) progressed to a low-quality classification (poor or unacceptable) during follow-up evaluations. Specifically, progression to poor or unacceptable classification occurred in 4 of 34 corneas (11.8%) initially classified as excellent, 92 of 670 corneas (13.7%) initially classified as good, and 36 of 108 corneas (33.3%) initially classified as fair. Notably, one-third of the tissues initially categorized as fair progressed to a low-quality classification over time.

The other variables also did not influence the progression of tissues to the lower-quality group, that is, those classified as poor or unacceptable, as shown in Table 2.

**Table 2:** Processing data of donated corneas for transplants followed at the Eye Bank. Campo Grande, MS—Brazil, 2025 (n = 844)

Variables		Corneal classification		*P*
		Progression to a lower classification	No progression to a lower classification	
*Median – IQR*				
Transport box temperature at the exit of the EB	3ºC (2ºC—3ºC)	–	–	0.676^¶^
Transport box temperature at the enucleation site	4ºC (3ºC—4ºC)	–	–	0.747^¶^
Transport box temperature upon return to the EB	5ºC (4ºC—5ºC)	–	–	0.445^¶^
	n*(%)			
*Refrigerated body*				0.109^*^
No	177 (41.7%)	46	308	
Yes	246 (58.3%)	90	400	
*Person responsible for the interview*				0.963^**^
Nurse	94 (22.3%)	30	158	
Nurse technician	317 (74.6%)	106	524	
Others	13 (3.1%)	04	22	
*Responsible for enucleation*				0.681^*^
Nurse	57 (13.5%)	19	95	
Nurse technician	365 (86.5%)	118	612	
*Responsible for processing*				0.500^**^
Nurse	09 (2.1%)	00	18	
Nurse technician	408 (96.7%)	135	681	
Doctor	05 (1.2%)	02	08	
*Place of enucleation*				0.502^**^
State capital hospital	284 (67.3%)	83	485	
Hospital in the countryside of the state	09 (2.1%)	03	15	
IMOL	82 (19.4%)	30	134	
SVO	35 (8.3%)	16	54	
PAX	09 (2.2%)	04	14	
UPA	03 (0.7%)	01	05	
*Preservation medium*				0.873^*^
Eusol-C	138 (32.7%)	44	232	
Optisol-GS	284 (67.3%)	93	475	

n*, Number of donors by classification and bilaterality of eyes; EB, Eye Bank; IQR, Interquartile Range; IMOL, Institute of Legal Medicine and Dentistry; SVO, Death Verification Service; PAX, Funeral Assistance Services; UPA, Emergency Care Unit; *,chi-square test; **, Fisher’s exact test; ^¶^ Mann–Whitney U test

To assess whether the 13 morphological aspects used in corneal quality staging showed changes between the first and second evaluations, the Wilcoxon test for paired samples was applied. This analysis maintained the original nature of the outcome variable, corneal tissue quality, without grouping. That is, the final classification scores of the corneas assigned by EB professionals were considered on an ordinal scale from 0 to 4. Through this test, it was evident that the tissues showed significant changes in the classifications assigned by EB professionals during the two evaluations using the slit lamp.

The effect size was evaluated through the biserial correlation, which revealed a strong negative correlation for criteria such as epithelial exposure, epithelial defect, subepithelial opacity, loss of endothelial cells, senile arc, pterygium, scarring, and specular reflection, with values close to  − 1.

These results indicate that, for the morphological criteria assessed, scores were generally lower (indicating better tissue quality) in the first evaluation. In the second evaluation, a trend of increasing scores was observed, suggesting poorer tissue classifications at this subsequent assessment point (Table 3).

**Table 3:** Morphological aspects of corneas donated for transplants and their relationship with tissue classification between the first and second evaluation moments. Campo Grande, MS—Brazil, 2025 (n = 844)

Variables	First evaluation -Median (IQR)	Second evaluation—Median (IQR)	*p*-value	Rank biserial correlation
Epithelium exposure	0 (0–1)	1 (0–2)	** < 0.001**	–0.842
Epithelial defect	0 (0–0)	0 (0–1)	** < 0.001**	–0.911
Subepithelial opacity	0 (0–0)	0 (0–1)	** < 0.001**	–0.895
Stromal edema	1 (1–1)	1 (1–1)	** < 0.001**	0.298
Stromal striae	0 (0–1)	0 (0–1)	** < 0.001**	–0.320
Stromal infiltrate	0 (0–0)	0 (0–0)	** < 0.001**	–0.527
Descemet’s membrane folds	1 (1–1)	1 (1–2)	** < 0.001**	–0.520
Endothelial cell loss	0 (0–0)	0 (0–1)	** < 0.001**	–0.889
Endothelial guttata	0 (0–0)	0 (0–0)	**0.005**	–0.283
Senile arc	0 (0–1)	1 (0–2)	** < 0.001**	–0.874
Pterygium	0 (0–0)	0 (0–0)	** < 0.001**	–0.869
Scars	0 (0–0)	0 (0–0)	** < 0.001**	–0.974
Specular reflection	0 (0–0)	0 (1–1)	** < 0.001**	–0.883
Overall score	1 (1–1)	1 (1–2)	** < 0.001**	–0.386

n*, Sample size; IQR, Interquartile Range; *, Wilcoxon test for paired samples

Bold values indicate statistical significance

It is important to emphasise that, although the Wilcoxon test for paired samples identified statistically significant differences in several variables, the medians and IQRs remained unchanged between the two evaluations for some of them.

This occurs because the Wilcoxon test is sensitive to systematic changes in the distributions of paired samples, even when measures of central tendency, such as the median, remain stable. Thus, despite stable medians and IQRs in some variables, a consistent pattern of classifications with higher scores (indicating lower tissue quality) in the second evaluation was demonstrated both by the rejection of the null hypothesis (that there is no difference between the two time points) and by the large effect sizes observed.

To verify whether the changes occurred independently of the storage medium, a stratified analysis was performed. The results indicated a similar pattern in both groups, with a tendency toward higher scores in the second evaluation, regardless of the preservation solution used. However, small differences were observed in the stromal criteria (subepithelial opacity, stromal edema, and stromal infiltrate), which may reflect the osmotic properties inherent to each solution (Table 4).

**Table 4:** Morphological aspects of corneas donated for transplantation and their relationship with tissue classification between the first and second evaluation moments, stratified by storage medium. Campo Grande, MS, Brazil, 2025 (n = 844)

	Optisol-GS (n = 568)			Eusol- C (n = 276)		
Variables						
	First evaluation (IQR)	Second evaluation (IQR)	*p- value* (effect size)	First evaluation (IQR)	Second evaluation (IQR)	*p- value* (effect size)
Epithelium exposure	0 (0–1)	1 (0–1)	**< 0.001** (0.795)	0 (0–1)	1 (0–2)	**< 0.001** (0.921)
Epithelial defect	0 (0–0)	0 (0–1)	**< 0.001** (0.915)	0 (0–0)	0 (0–1)	**< 0.001** (0.904)
Subepithelial opacity	0 (0–0)	0 (0–1)	**< 0.001** (0.937)	0 (0–0)	0 (0–0)	**< 0.001** (0.675)
Stromal edema	1 (1–1)	1 (0–1)	**< 0.001** (0.399)	1 (1–1)	1 (1–1)	0.821 (0.027)
Stromal striae	0 (0–0)	0 (0–1)	**< 0.001** (0.581)	1 (0–1)	1 (0–1)	0.025 (0.022)
Stromal infiltrate	0 (0–0)	0 (0–0)	**0.003** (0.674)	0 (0–0)	0 (0–0)	0.132 (0.333)
Descemet’s membrane folds	1 (1–1)	1 (1–2)	**< 0.001** (0.423)	1 (1–1)	1 (1–2)	**< 0,001** (0.709)
Endothelial cell loss	0 (0–0)	0 (0–0)	**< 0.001** (0.935)	0 (0–0)	0 (0–1)	**< 0,001** (0.754)
Endothelial guttata	0 (0–0)	0 (0–0)	**< 0.001** (0.574)	0 (0–0)	0 (0–0)	**0,004** (0.349)
Senile arc	0 (0–1)	1 (0–2)	**< 0.001** (0.830)	0 (0–1)	1 (0–1)	**< 0.001** (-0.955)
Pterygium	0 (0–0)	0 (0–0)	**< 0.001** (0.929)	0 (0–0)	0 (0–0)	**0.002** (-0.689)
Scars	0 (0–0)	0 (0–0)	**< 0.001** (0.990)	0 (0–0)	0 (0–0)	**< 0.001** (-0.779)
Specular reflection	0 (0–0)	0 (0–0)	**< 0.001** (0.862)	0 (0–0)	1 (0–1)	**< 0.001** (-0.931)
Overall score	1 (1–1)	1 (1–2)	**< 0.001** (0.321)	1 (1–1)	1 (1–2)	**< 0.001** (-0.551)

n*, Sample size; IQR, Interquartile Range; * = Wilcoxon test for paired samples; Effect size, Rank Biserial Correlation

Bold values indicate statistical significance

## Discussion

The donation and transplantation process involves several factors that directly impact the success of keratoplasties. This cohort study highlights that when processes are adequately standardized, the quality of the corneal tissue is maintained without significant changes between the first and second evaluations that would lead the tissue to be classified in the group of worst-quality corneas, which would compromise its indication for therapeutic use.

This stability was observed regardless of the medium-term hypothermic storage solution used, whether Optisol-GS or Eusol-C. The results showed a very low relative risk of the tissues evolving to the worst-quality cornea group (until the fourth day of storage), with a probability of 1% without statistical significance.

These findings are supported by studies indicating that these media can be alternative solutions to each other, capable of meeting the surgeons’ needs in keratoplasty (Ramini et al. 2025; Kanavi et al. 2015). However, an important detail observed in the literature is the indication that Optisol-GS tends to maintain a thinner tissue compared to Eusol-C, which would be preferred in lamellar keratoplasties (Ramini et al. 2025).

This aspect may be related to their formulations, as Optisol-GS contains chondroitin, which is an essential component for preventing corneal edema since it acts as a hyperosmotic agent, promoting the reduction of corneal swelling (Ramini et al. 2025; Tran et al. 2023).

Furthermore, the results can be explained by the fact that, in the service where the study was conducted, corneas are evaluated within a short timeframe of less than seven days. Established evidence in the literature indicates that one of the main factors for maintaining tissue quality is reduced storage time in EBs, whereas periods longer than seven days are associated with a significant increase in endothelial cell loss (Gimenes et al. 2022).

These findings suggest that although tissues can be stored for up to 14 days with suitability for transplantation, shorter storage periods may be more effective in preserving the quality of corneas in the group of tissues suitable for therapeutic use.

Since both media exhibited similar characteristics for corneal preservation, it is necessary to advance the production of scientific evidence and to consider conducting economic analyses to assess the financial impact of the two media on the services.

In particular, cost-minimization studies can be valuable tools in this process, as they analyze the economic advantages of health technologies that demonstrate similar efficacy (Brasil, 2014), such as the preservatives evaluated in this study, which can support more assertive and sustainable health decisions.

Another relevant finding of this study was that, for this sample, sociodemographic and processing conditions did not influence the progression of tissues initially classified as excellent, good, or fair to the group of corneas considered poor or unsuitable for transplantation—that is, those of lower quality that would no longer be allocated for transplant use. These findings reinforce that corneas initially assessed as higher quality remained stable within this classification.

Although the grouped results showed a low progression of corneas classified in the best quality group to the worst quality group between the first and second evaluation, a consistent trend of higher classification scores in the second evaluation was identified, and this difference was confirmed by the Wilcoxon test for paired samples.

Upon identifying discrepancies in the scores assigned to the corneal tissues at the two evaluation time points, it becomes evident that these differences may be related to human factors and the inherent subjectivity of slit-lamp evaluation. Since the final classification of the tissue is determined exclusively by the ophthalmologists at the EB, the clinical experience and diagnostic accuracy of these professionals may directly influence the quality assigned to the tissue at this stage.

Although the nurse and the ophthalmologists at the EB follow the same classification criteria, as presented in Box 1, the evaluators’ level of experience and individual differences in visual perception may influence the scores assigned to the tissue (Cruz et al. 2022). This variability becomes even more apparent when each of the thirteen morphological criteria is analyzed separately, as certain characteristics that should not be affected by storage conditions, such as arcus senilis, pterygium, and scarring, tended to receive higher scores (indicating lower tissue quality) during the second evaluation.

The results of the stratified analysis reinforce the hypothesis of interobserver variability. Although small differences were observed in stromal criteria, such as subepithelial opacity, stromal edema, and stromal infiltrate, possibly related to the compositional characteristics of the preservation media, these variations were subtle and did not affect the overall tissue classification. Thus, the findings suggest that the discrepancies between evaluations are more likely attributable to interobserver subjectivity than to the storage medium itself.

It is also important to highlight that environmental influences such as time allocated for evaluations, temperature, or equipment variation did not occur during the study period.

To support the training of nurses performing slit-lamp evaluations in EBs in Brazil, the Pan-American Association of EBs previously offered training and certification programs (Battle 2002). The nurse responsible for corneal evaluations at the EB analyzed in this study was certified through one of these programs. These training programs aimed to qualify professionals for all stages of activities within the EB; however, the association discontinued its activities in Brazil during the COVID-19 pandemic.

Following the discontinuation of these activities, EBs professionals across different regions of the country have sought to fill the resulting gap by developing local continuing education initiatives aimed at training and qualifying new staff. However, these efforts remain fragmented and regionally constrained, lacking the national reach, methodological consistency, and institutional standardization that had previously been ensured by the Pan-American Association of EBs certification programs.

Since different professionals perform evaluations between the first and second moments, it emphasizes the importance of adopting measures that minimize subjectivity between evaluations. Continuous training and a calibration process among evaluators are essential to ensure that the two evaluation moments follow similar standards (Cruz et al. 2022).

Additionally, the adoption of equipment that allows for objective evaluation, such as specular microscopes, is a fundamental measure to reduce subjectivity. Although this equipment is considered the gold standard for corneal quality classification due to its ability to provide endothelial cell counts, it is a high-cost piece of equipment (Vilela et al. 2024), and not all EBs have the financial resources to acquire it.

It is important to highlight that EBs in Brazil may operate under different organizational and funding models, including public services, hybrid (public–private) institutions, in which transplantation is provided free of charge but part of the service provision is performed by third parties, and non-profit philanthropic institutions. Due to these differences in funding, the resources and infrastructure available vary among the 46 EBs in the country. As a result, some services have access to multiple tissue evaluation technologies, such as specular microscopy, optical coherence tomography, and slit-lamp biomicroscopy, whereas others, such as the service evaluated in this study, rely exclusively on the latter technology.

In general, Brazilian EBs follow the national transplant legislation in force since 1997 (Brasil 1997; Brasil, 2025). Each state that has an EB is supervised by a state transplant system, which is responsible for coordinating with the National Transplant System. Regarding the operation of EBs, the process occurs as follows: after the confirmation of the death of a potential donor, a team is activated to conduct an interview with the family members. This process follows the opt-in model (Silva et al. 2024), in which, regardless of the donor’s prior manifestation during life, the final decision rests with the family.

With family authorization, a specialized team performs tissue procurement, which may occur through enucleation or in situ excision. Subsequently, the material is transported in a thermal container under controlled temperature conditions to the EB. At the facility, the cornea is processed, evaluated, stored, and made available for transplantation (ANVISA, 2025).

In EBs with access to different technologies, corneal evaluation is performed through a combination of methods, generally at two distinct time points. In contrast, in EBs that do not have equipment allowing quantitative assessments, the analysis is entirely qualitative. After evaluation, the tissue may be classified as suitable for optical transplantation, suitable for tectonic transplantation, or unsuitable for transplantation — in the latter case, it may either be discarded or allocated for research and validation of the bank’s internal processes.

The literature is scarce regarding the access of EB in developing countries to such equipment, but there is an understanding that using this method to assign tissue quality yields better results, as tissues with higher endothelial cell densities are associated with keratoplasties that have a longer survival rate (Vasiliauskaitė et al. 2021; Chaussard et al. 2022).

This study presents the limitations related to the data source used for obtaining information. The endothelial cell count through specular microscope evaluation could not be assessed, as the service where the study was conducted does not have this equipment, making the tissue quality assignment entirely qualitative.

Additionally, the donation forms, which served as the standard instrument for tissue procurement by the banks, do not include information on the surgical history and comorbidities of the donor individuals, limiting the analysis of relevant clinical variables for the context.

Overall, it is important to highlight that the tendency toward higher scores in the second evaluation did not result in a relevant impact on the overall classification of corneas for transplantation, suggesting that the observed changes were subtle, although detectable by the statistical tests employed, which can identify systematic patterns of difference between evaluation moments.

However, the design of this study was not specifically developed to investigate the reliability of these assessments. Therefore, diagnostic accuracy studies with samples specifically planned for this purpose, including interobserver (different evaluators) and intraobserver (same evaluator) assessments at different time points, combined with reliability tests designed to measure the consistency of these evaluations, may provide more robust evidence and contribute to the exploration of a topic that remains poorly discussed in the literature on EB assessments.

Finally, as a perspective for future studies, the potential benefits of longer exposure periods of previously refrigerated vials to room temperature before evaluation should be investigated, considering that the 10-min interval adopted in the EB protocol may not be sufficient to achieve complete thermal equilibration between the vial and the surrounding environment. Extending this thermal stabilization period may potentially improve corneal endothelial visualization during biomicroscopic assessment. Previous studies have suggested that warming donor corneas to temperatures closer to physiological conditions may enhance endothelial visualization quality (Tran et al. 2017).

## Conclusion

The results of this study provided valuable indicators that reinforce the available evidence that both Eusol-C and Optisol-GS are viable media for use in EBs without a significant impact on the progression of tissues to poorer quality groups.

However, it was observed that tissues stored in the EB tended to progress to higher scores in the second evaluation, indicating that after 44 h and 48 min (interquartile range: 33 h and 0 min to 63 h and 30 min), the tissues were classified as having lower quality compared with the first evaluation performed. This difference may be related to the subjectivity of slit-lamp evaluations, since even when evaluators adopt the same assessment criteria, they may have different perceptions of the quality of the evaluated corneas.

Therefore, specific studies are needed to investigate the diagnostic precision and accuracy of slit-lamp evaluations, as well as the impact of interobserver subjectivity on tissue quality classification. The findings of this study may contribute to strengthening the available evidence that both hypothermic media represent viable alternatives for corneal storage for transplantation, particularly in services that exclusively use hypothermic preservation solutions.

## Data Availability

The datasets generated during and/or analysed during the current study are available from the corresponding author on reasonable request.
